# Dermatitis Herpetiformis Revealing Acute Lymphoblastic Leukemia in a Child: A Case Report

**DOI:** 10.7759/cureus.106735

**Published:** 2026-04-09

**Authors:** Mariam Lagrine, Rabiy Elqadiry, Houda Nassih, Aicha Abourrahouat, Imane Aitsab

**Affiliations:** 1 Department of Pediatrics B, Mother and Child Hospital, Centre Hospitalo-Universitaire Mohammed VI de Marrakech, Marrakech, MAR; 2 Department of Pediatrics, Laboratoire de Recherche l'Enfance, la Sante et le Développement Durable, Marrekech, MAR; 3 Faculty of Medicine and Pharmacy, Cadi Ayyad University, Marrakech, MAR

**Keywords:** acute lymphoblastic leukemia, celiac disease, dermatitis herpetiformis, gluten-related disorders, vesiculobullous eruption

## Abstract

A two-year-and-nine-month-old girl was referred for widespread bullous and herpetiform skin lesions associated with limb edema and asthenia, evolving over approximately seven months. The initial clinical evaluation raised suspicion of bullous systemic lupus erythematosus based on cutaneous findings and abnormal blood tests, including proteinuria, hypoalbuminemia, and low complement C4. Skin histopathology showed dermal edema with a neutrophilic infiltrate, and direct immunofluorescence revealed granular IgA and C3 deposits along the dermoepidermal junction, findings diagnostic of dermatitis herpetiformis (DH). Serologic tests showed normal total IgA, negative anti-transglutaminase and anti-endomysial antibodies, and positive anti-gliadin antibodies. Jejunal biopsy showed no villous atrophy. The diagnosis of celiac disease was not made, as anti-gliadin antibodies are nonspecific and were not supported by confirmatory serologic or histologic evidence. Hematologic reassessment revealed pancytopenia and circulating blasts, and bone marrow aspiration confirmed acute lymphoblastic leukemia (ALL). The patient was referred to pediatric oncology for treatment of her leukemia. A gluten-free diet was initiated specifically for the management of DH, which is a gluten-sensitive dermatosis. The patient showed a favorable clinical evolution with the resolution of skin lesions. This case highlights an unusual association between DH and pediatric ALL. It underscores the importance of maintaining a broad differential diagnosis that includes hematologic malignancies when evaluating children with atypical or treatment-refractory dermatologic presentations, even when a specific dermatologic diagnosis, such as DH, has been established.

## Introduction

Dermatitis herpetiformis (DH) is an autoimmune blistering disorder recognized as the cutaneous manifestation of gluten-sensitive enteropathy. Its pathogenesis is triggered by gluten, which leads to the production of IgA antibodies against tissue transglutaminase. In DH, the primary target antigen is epidermal transglutaminase (TG3). The resulting immune complexes deposit as IgA in the dermal papillae, recruiting neutrophils and causing the characteristic rash. Detection of these granular IgA deposits via direct immunofluorescence is the gold standard for diagnosing DH and distinguishing it from other vesiculobullous disorders. DH is more common in adults but can occasionally affect children with underlying genetic susceptibility [1,2].

The relationship between DH and celiac disease is well established; however, the spectrum of gluten-related disorders extends beyond overt enteropathy. Patients may present with potential celiac disease, defined by the presence of gluten-dependent symptoms or DH with positive serology, yet without demonstrable villous atrophy on jejunal biopsy [3]. This case exemplifies such a presentation, where cutaneous findings preceded and ultimately guided the diagnosis of gluten sensitization.

The coexistence of DH with hematologic malignancies is exceptionally uncommon, particularly in the pediatric population. While adults with long-standing celiac disease have an increased risk of lymphoproliferative disorders, the association between DH and acute lymphoblastic leukemia (ALL) in a child is exceedingly rare, with few cases reported in the literature [4,5]. When DH presents alongside systemic symptoms such as pallor, edema, or fatigue, clinicians must maintain a broad differential diagnosis that includes autoimmune and hematologic disorders.

We present a case of a two-year-and-nine-month-old girl in whom investigation of widespread vesiculobullous lesions ultimately revealed both DH and underlying ALL. This case highlights the critical importance of considering systemic and malignant conditions in children with atypical or treatment-refractory dermatologic presentations and underscores the value of multidisciplinary collaboration between dermatology, gastroenterology, and hematology in achieving timely diagnosis and optimal outcomes.

## Case presentation

**Figure 1:**
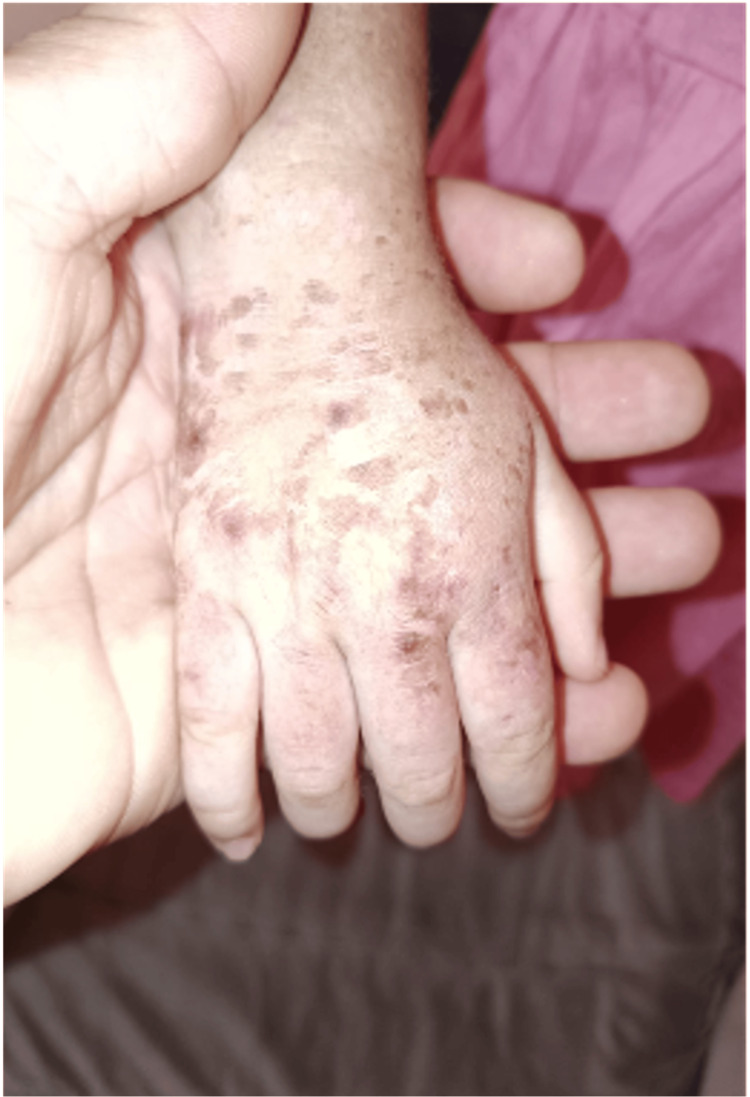
Crusted post-bullous lesions on the legs and feet, with hypopigmented scars on the trunk and limbs. Nikolsky’s sign was negative.

A two-year-and-nine-month-old girl was referred by a general pediatrician for evaluation of widespread tense vesiculobullous lesions involving the face, neck, trunk, and extensor surfaces of the limbs, evolving over several months. The lesions were described as intensely pruritic and progressed from vesiculobullous to crusted lesions, with associated hypopigmented scarring. Mucous membrane involvement (oral and genital) was noted during the initial evaluation (Figure 1). The clinical presentation was also marked by progressive limb edema, intermittent fever, and asthenia, which had been ongoing for approximately seven months.

During the first month of symptoms, the mucocutaneous findings and abnormal laboratory results, including hypoalbuminemia and proteinuria, raised suspicion for bullous systemic lupus erythematosus. The patient was initially started on corticosteroids (1 mg/kg/day).

At admission to our department one month later, the child appeared pale and underweight (weight: 11.5 kg, -3 SD; height: 86 cm, median). Vital signs were stable. Physical examination showed no hepatosplenomegaly or cardiac abnormalities. Laboratory findings revealed normocytic anemia (hemoglobin 10.5 g/dL, mean corpuscular volume 79 fL), a proteinuria/creatininuria ratio of 0.55, a serum albumin of 30 g/L, a platelet count of 161,000/mm³, and a leukocyte count of 6,500/mm³. Renal and liver functions were normal. Complement analysis showed normal C3 and decreased C4 (0.03 g/L) (Table 1).

**Table 1:** Laboratory findings of the patient with reference values

Parameter	Result	Reference range
Hemoglobin	10.5 g/dL	11-14 g/dL
Mean corpuscular volume	79 fL	75-87 fL
Platelets	161,000/mm³	150,000-450,000/mm³
Leukocytes	6,500/mm³	5,000-15,000/mm³
Proteinuria/creatininuria ratio	0.55	<0.2
Albumin	30 g/L	35-50 g/L
Complement C3	Normal	0.9-1.8 g/L
Complement C4	0.03 g/L	0.1-0.4 g/L
Uric acid	32.5 mg/dL	2-7 mg/dL
Lactate dehydrogenase	591 IU/L	140-280 IU/L

**Figure 2:**
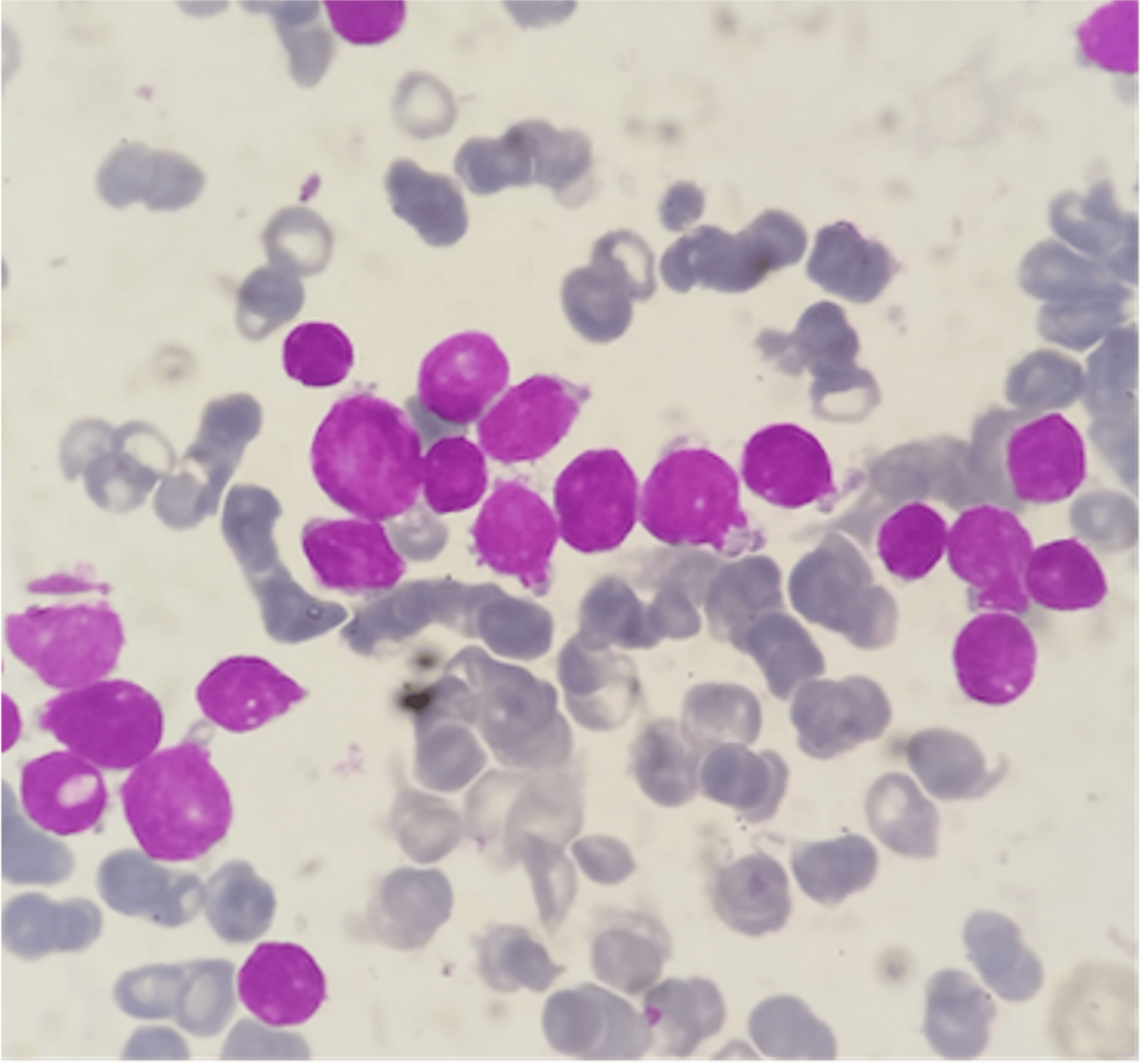
Bone marrow aspirate showing small blasts with a high nuclear-to-cytoplasmic ratio, fine chromatin, and scant basophilic cytoplasm

The child did not show full improvement with corticosteroids. While the steroids reduced the swelling, the blister-like skin lesions and systemic symptoms persisted, prompting further evaluation. In light of this progression, a repeat blood count revealed worsening anemia, thrombocytopenia, and leukopenia. Tumor lysis parameters were elevated. Bone marrow aspiration and biopsy confirmed a diagnosis of ALL (Figure 2).

**Figure 3:**
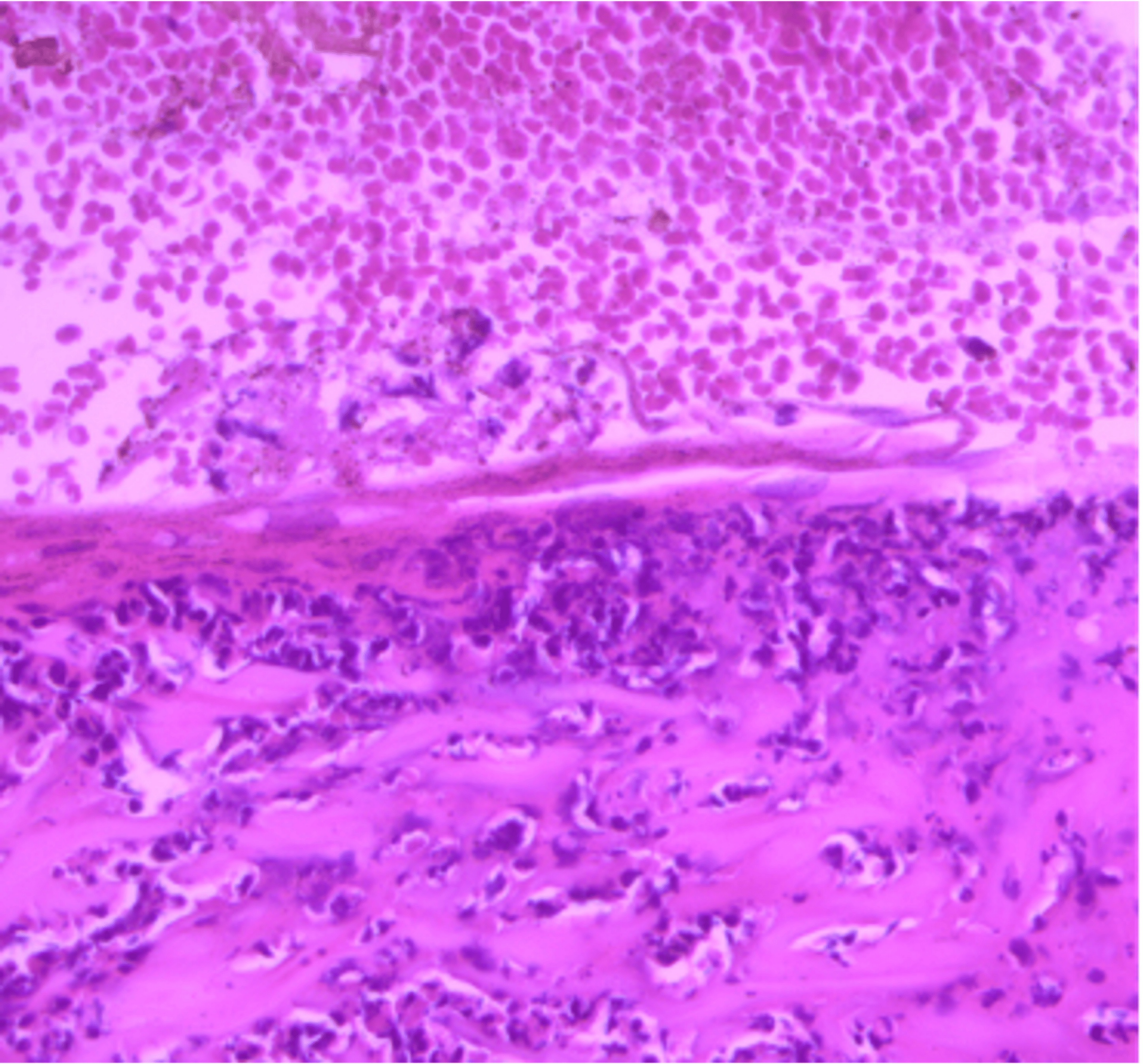
H&E staining (×40) showing a subepidermal blister with a neutrophilic infiltrate in the dermal papillae

**Figure 4:**
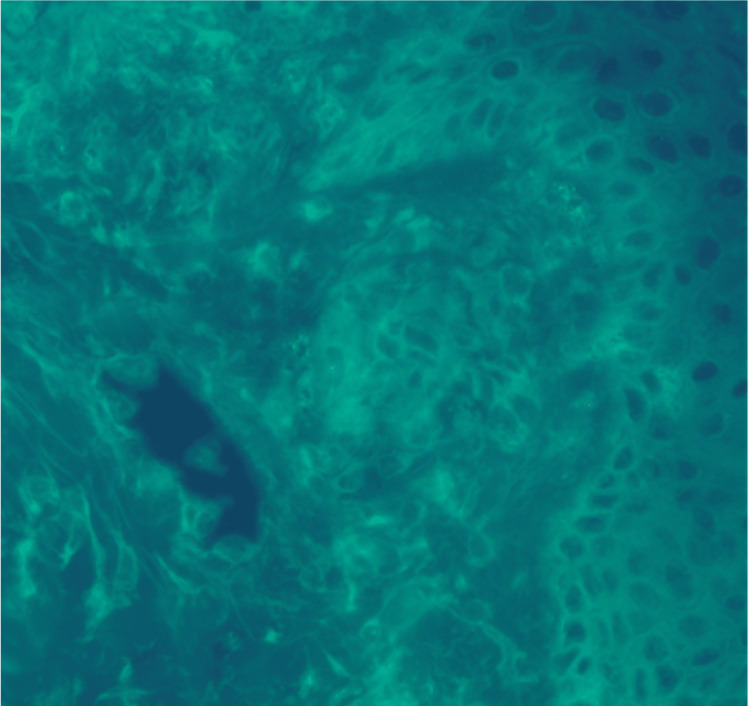
Direct immunofluorescence showing granular IgA deposits in the dermal papillae

**Figure 5:**
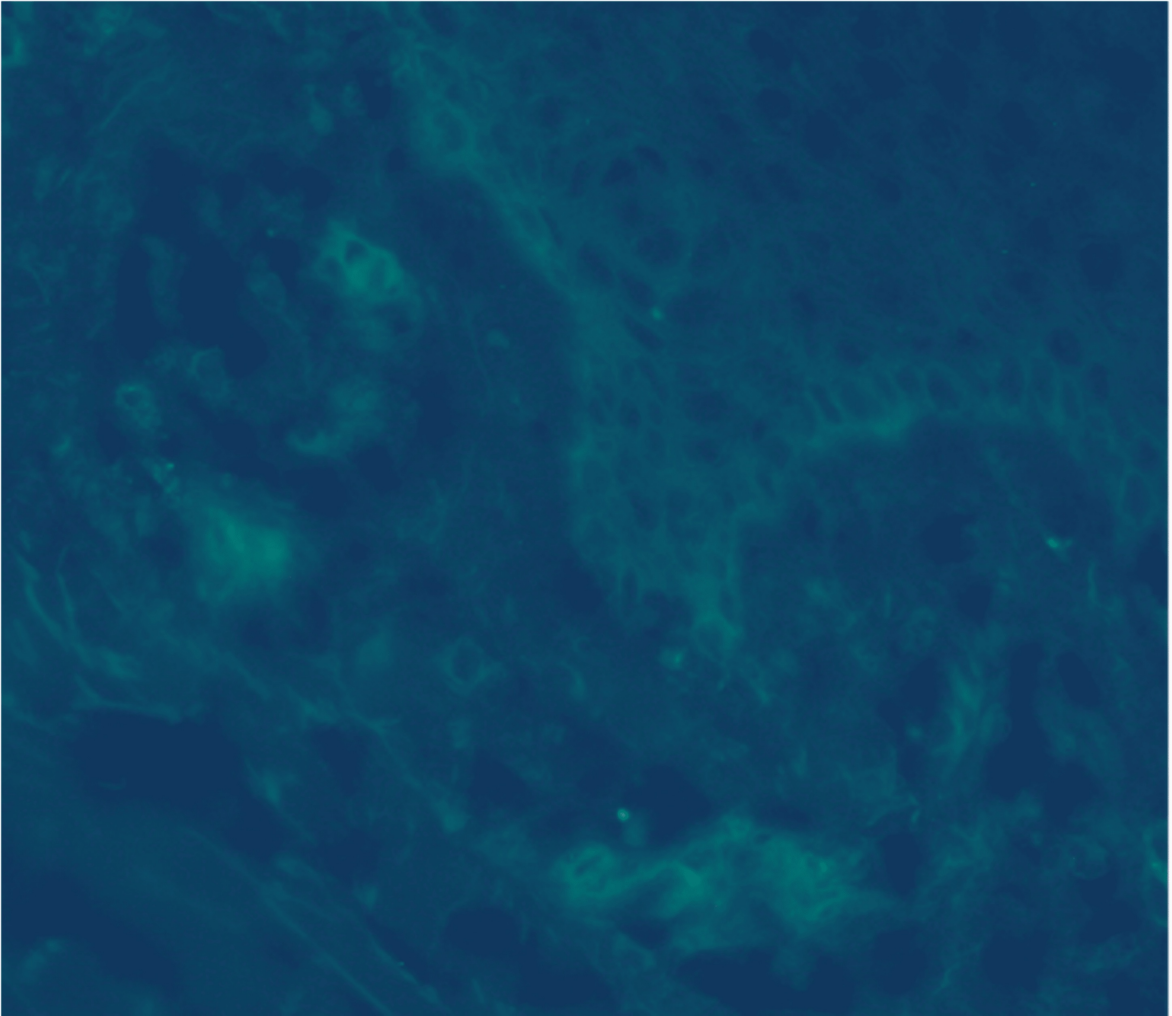
Direct immunofluorescence showing granular C3 deposits in the dermal papillae

A skin biopsy was performed. Histopathology revealed subepidermal blister formation with a neutrophilic infiltrate in the dermal papillae (Figure 3). Direct immunofluorescence revealed deposits of IgA (Figure 4) and C3 (Figure 5) at the dermoepidermal junction, findings diagnostic of DH.

Celiac serology showed normal total IgA (1.61 g/L), negative anti-transglutaminase IgA and anti-endomysial antibodies, and positive anti-gliadin antibodies. Jejunal biopsy revealed intact villi. Based on these findings, the diagnosis of celiac disease was excluded, as anti-gliadin antibodies are nonspecific and were not supported by confirmatory serologic or histologic evidence.

The patient was diagnosed with DH in the setting of ALL. She was referred to pediatric oncology for treatment of her leukemia. A gluten-free diet was initiated specifically for the management of DH, which is a gluten-sensitive dermatosis, regardless of the absence of confirmed celiac disease.

## Discussion

This case illustrates the diagnostic complexity of multisystem disorders in pediatric patients. The initial presentation, suggestive of bullous systemic lupus erythematosus, ultimately proved to be DH, as revealed through histopathology and immunofluorescence findings. Although DH is typically linked to celiac disease, its coexistence with hematologic malignancy, particularly ALL, is extremely uncommon [3]. Previous reports have described associations between gluten-sensitive enteropathy and lymphoproliferative disorders, mostly in adults [4,5]. Pediatric cases remain exceptional.

The serologic profile of positive anti-gliadin antibodies, negative transglutaminase and endomysial antibodies, and normal jejunal architecture aligns with potential celiac disease, a state of gluten sensitization without intestinal atrophy. The cutaneous IgA deposition observed here reinforces the concept of extra-intestinal gluten-related autoimmunity [6].

Chronic immune activation related to gluten exposure might contribute to systemic immune dysregulation, which in genetically predisposed individuals could favor oncogenic processes [7,8]. While causal links remain speculative, this case highlights the possibility that gluten-related immune imbalance may play a role in pediatric hematologic disease.

From a gastroenterologic perspective, children with potential celiac disease require long-term surveillance to monitor possible evolution toward overt enteropathy. This case, therefore, emphasizes the importance of multidisciplinary collaboration between dermatology, gastroenterology, and hematology to ensure early recognition of systemic manifestations and optimal patient outcomes.

The coexistence of ALL and DH in this child suggests a possible link between immune dysregulation and malignancy, although causality cannot be inferred. Chronic immune activation related to gluten exposure has been associated with lymphoproliferative disorders in adults, but pediatric evidence remains scarce [9]. This case reinforces the importance of considering systemic immune dysfunction in children with atypical dermatologic presentations. Clinicians should maintain diagnostic vigilance when therapeutic responses are incomplete and promptly investigate potential hematologic or autoimmune comorbidities.

## Conclusions

Pediatric patients presenting with vesiculobullous eruptions and systemic symptoms require a broad differential diagnosis, including autoimmune and hematologic disorders. This case highlights a rare coexistence of DH and ALL. The diagnosis of DH was confirmed by direct immunofluorescence, while celiac disease was excluded based on negative specific serology and normal jejunal biopsy. A gluten-free diet was initiated for the management of the DH. Clinicians should maintain diagnostic vigilance when therapeutic responses are incomplete and promptly investigate potential underlying systemic conditions, including hematologic malignancies, in children with atypical dermatologic presentations.
